# Impact of Artificial Intelligence on Nursing Students’ Attitudes toward Older Adults: A Pre/Post-Study

**DOI:** 10.3390/nursrep14020085

**Published:** 2024-04-29

**Authors:** Anne White, Mary Beth Maguire, Austin Brown, Diane Keen

**Affiliations:** 1 Wellstar School of Nursing, Kennesaw State University, Kennesaw, GA 30144, USA; mbm2332@gmail.com (M.B.M.); dkeen2@kennesaw.edu (D.K.); 2 School of Data Science and Analytics, Kennesaw State University, Kennesaw, GA 30144, USA; abrow708@kennesaw.edu

**Keywords:** artificial intelligence, simulation training, aging, geriatrics, attitudes survey

## Abstract

As the global population ages, nurses with a positive attitude toward caring for older adults is crucial. However, studies indicate that nursing students often exhibit negative attitudes toward older adults. This study aimed to determine if a three-phased educational intervention significantly improved nursing students’ attitudes toward older adults. A pre/post-test study design was used to measure the change in nursing students’ attitudes toward older adults, as measured by the UCLA Geriatrics Attitudes Survey, after participating in an Artificial Intelligence in Education learning event (*n* = 151). Results indicate that post-intervention scores (M = 35.07, SD = 5.34) increased from pre-intervention scores (M = 34.50, SD = 4.86). This difference was statistically significant at the 0.10 significance level (t = 1.88, *p* = 0.06). Incorporating artificial intelligence technology in a learning event is an effective educational strategy due to its convenience, repetition, and measurable learning outcomes. Improved attitudes toward older adults are foundational for delivering competent care to a rapidly growing aging population. This study was prospectively registered with the university’s Institutional Review Board (IRB) on 30 July 2021 with the registration number IRB-FY22-3.

## 1. Introduction

As the global population ages, the demand for competent healthcare professionals with a positive attitude toward caring for older adults becomes increasingly crucial. Nurses hold a critical role in providing high-quality care to this vulnerable population. However, studies have indicated that nursing students often exhibit negative attitudes toward older adults, which can significantly impact their future practice of caring for older adults [1,2].

Recent reports have highlighted the need to improve the quality of geriatric care healthcare professionals provide, specifically emphasizing nursing education [3]. It has been observed that nursing students often harbor ageist attitudes, negative stereotypes, and limited knowledge about the unique healthcare needs of older adults [4]. These attitudes can hinder effective communication, compassionate care, and the overall well-being of older adults [5,6]. Consequently, efforts to address these attitudes and promote positive perceptions of senior care are essential in preparing a skilled nursing workforce to meet the demands of our aging population.

Ageism, defined as prejudice or discrimination against individuals based on age, remains a prevalent issue in healthcare settings, including nursing education [7]. Negative stereotypes and attitudes toward older adults can undermine the quality of care provided to this vulnerable population. A study by Allen et al. found that nursing students often held ageist beliefs, perceiving older adults as less capable, less deserving of care, and having limited potential for recovery [8]. Such attitudes can impact patient outcomes and the overall healthcare experience for older adults [9]. Therefore, addressing ageism within nursing education is imperative to develop a compassionate and person-centered approach to senior care.

Attitudes of healthcare professionals, particularly nurses, greatly influence the quality of care delivered to older adults. Positive attitudes toward aging and geriatric care are associated with better patient outcomes, increased patient satisfaction, and improved interprofessional collaboration [5]. Conversely, negative attitudes and stereotypes can result in suboptimal care, inadequate communication, and decreased patient well-being [4]. A systematic review by Burns et al. revealed that nursing students who often exhibited negative attitudes toward older adults were linked to insufficient exposure to geriatric nursing and a limited understanding of the aging process [10]. Thus, fostering positive attitudes among nursing students is essential to improve the overall quality of care for older adults and address the existing attitude-related barriers to effective geriatric nursing practice.

Educational interventions have shown promise in positively influencing nursing students’ attitudes toward caring for older adults. Several authors have used geriatric simulation exercises and reflective discussions to expose healthcare professions to older adults. The intervention significantly improved empathy toward aging and increased confidence in providing geriatric care [11,12]. An integrative review by Magan et al. found that past experiences with older adults and gerontology-focused teaching strategies effectively diminished ageist stereotypes and fostered positive attitudes and perceptions of older adults [12]. Similarly, a systematic review by Shirey et al. demonstrated the efficacy of educational interventions in improving nursing students’ knowledge, skills, and attitudes related to geriatric nursing [13].

The literature is replete with evidence regarding the impact of nurses’ attitudes when caring for older adults. Contemporary nursing education may influence students’ attitudes by incorporating innovative technology. An emerging pedagogy type involves Artificial Intelligence in Education (AIED) [14]. Artificial intelligence is the use of computers and machines to mimic the problem-solving skills of the human mind [15]. Therefore, this study aimed to determine if a three-phased AIED intervention significantly improved nursing students’ attitudes toward older adults.

## 2. Materials and Methods

### 2.1. Theoretical Framework

The Experiential Learning Theory was the theoretical framework to support this study. Kolb described it as a four-stage learning cycle: concrete experience (CE), reflective observation (RO), active experimentation (AE), and abstract conceptualization (AC). The CE is described as the experience, whereas the RO is the purposeful reflection after the experience. The AE represents the implementation of the learning, and the AC is learning from experience [16]. A key aspect of Kolb’s theory is that learning is not a linear process but a continuous cycle. Learners may revisit stages multiple times to refine their understanding and develop their skills.

### 2.2. Design

The researchers used a pre/post-test study design to measure the change in attitudes toward older adults among students enrolled in a senior-level community health nursing course in a baccalaureate program in the southeastern United States.

### 2.3. Sample Demographics

The population of interest is students enrolled in a senior-level community health nursing course in a baccalaureate program in the Southeastern United States. Since all students enrolled in the course were required to participate in the intervention as part of their course requirements, this implies that the sampling technique is non-probabilistic, the sample size relative to the population size is nearly 100%, and that attrition will not have a substantial role in the study. Further, since the sample size is almost the same as the size of the target population, the study is as maximally powered as is feasible since further participant recruitment was not possible. Thus, traditional power analysis techniques were deemed irrelevant for the present study. The sample demographics included the gender and age of the students enrolled in the community health class.

### 2.4. Inclusion and Exclusion Criteria

The inclusion criteria were all students enrolled in the community health nursing course. Exclusion criteria were incomplete datasets.

### 2.5. Instrument

The UCLA Geriatric Attitude Scale (GAS) was used to measure nursing students’ attitudes before and after the educational intervention [17]. The GAS is a widely used instrument that assesses attitudes toward aging and older adults, providing valuable insights into respondents’ prevailing attitudes and beliefs [17]. The instrument contains five positively and nine negatively worded statements rated on a scale from 1 (strongly disagree) to 5 (strongly agree), with higher scores indicating more positive attitudes toward aging. The internal reliability for the instrument was Cronbach’s alpha = 0.76 [10]. An alpha between 0.7 and 0.9 is considered acceptable.

### 2.6. Statistical Methods

Because the study aimed to determine if the AIED changed nursing student attitudes toward older adults, a paired means sample test was deemed appropriate. Traditionally, a paired means sample *t*-test is used in such cases. Still, as is well-known, the validity of this test depends upon the assumption of normality being reasonably met. Before performing the paired means sample *t*-test, both a visual and a testing method of evaluating normality were employed, with the former being a Quantile–Quantile plot and the latter being the classical Shapiro–Wilk test of normality. In addition to the *p*-value of the inferential test used, the effect size was also reported to contextualize the results better.

### 2.7. Intervention

The community health nursing course utilized a three-phased approach to the AIED event (Table 1). The learning activities focused on Millie Larsen, a National League for Nursing (NLN) Advanced Care for Seniors (ACES) unfolding case [18]. The case study was introduced in the class and served as the foundation for complimenting activities related to older adult care.

Phase One included assigned pre-class readings, 90-min classroom instruction, a review of the simulated patient’s medical record, and a 22-item pre-simulation knowledge survey regarding the information reviewed to validate the completion of the Phase One activity. Phase Two comprised an AI-driven, virtual, non-immersive simulation experience of Millie Larson in her home [19]. The AI-driven virtual simulation replicated a patient interview using advanced artificial intelligence and natural language processing technology. Faculty members programmed the simulator prompts to ensure accurate responses to inquiries and adherence to the desired learning outcomes. Students interacted with the virtual simulator on their personal computers. Leveraging their computer’s microphone, learners engaged in dialogue with the virtual patient. Each student was equipped with an individual account granting unrestricted access, free of charge, due to an in-kind grant. The virtual nature of the technology allowed for repeated practice sessions at their convenience to complete an abbreviated Outcome and Assessment Information Set Start of Care (OASIS SOC) [20]. Also, students drafted an email to a simulated multidisciplinary team describing the findings of the simulated patient encounter. Phase Three included completing an online reflection and debrief related to the simulated encounter and completing the NLN Simulation Design Scale instrument [21]. Due to the asynchronous nature of the learning event, students were given one week to complete the activities.

### 2.8. Ethical Aspects

The university’s Institutional Review Board (IRB) deemed the study exempt from review. Participants were informed that completing the learning activity was a course requirement. Additionally, all study-related data were de-identified and stored on password-protected computers.

## 3. Results

### 3.1. Sociodemographic of Sample

A total of 160 students enrolled in the course (N = 160), and only those who completed all data were included in the study (*n* = 151), or 94% of the population. Table 2 represents a summary of the demographic data. Most of the sample identified as female (90%) and were between 18 and 24 years old (59; 39%).

### 3.2. Instrument Reliability

The instrument’s internal reliability was Cronbach’s alpha = 0.76 for the pre-intervention scores and 0.78 for the post-intervention scores.

### 3.3. Mean Differences of Scores

Because the study aimed to determine if the AIED changed nursing student attitudes toward older adults, a paired sample *t*-test was used to compare the pre- and post-experience scores. Of note, only students with pre- and post-experience scores were included in the analysis (*n* = 151). Results indicate that post-intervention scores (M = 35.07, SD = 5.34) increased from pre-intervention scores (M = 34.50, SD = 4.86). This modest improvement (d = 0.15) was statistically significant at the 0.10 significance level (t = 1.88, *p* = 0.06). An item-by-item analysis is given in Table 3. To note, all statistical analyses were performed in the statistical software package R version 4.3.2.

## 4. Discussion

This study aimed to add to the existing literature by assessing the influence of an AIED event on nursing students’ feelings around ageism and attitudes toward caring for older adults. The findings reveal a significant increase in attitude scores after the AIED event. The results of this study support previous studies that identified education and intergenerational contact as effective interventions to combat ageism, thus improving attitudes toward older adults [6,9,10,22]. The outcomes propose that employing a multi-phased approach with a virtually simulated experience might overcome ageism in healthcare among baccalaureate nursing students.

The AIED event allowed undergraduate nursing students to experience a realistic encounter with an older adult, raising students’ awareness of their attitudes toward the geriatric population. This study’s results are like those of other studies that used simulation as an educational intervention to positively influence nursing students’ attitudes toward caring for older adults [11,12]. This study underscores the efficacy of the AIED event in challenging negative attitudes and promoting more positive perceptions of older adults among future nursing workforces.

Nurses need to be at the forefront of evolving simulation pedagogy to help progress technologies in a manner sensitive to inclusivity and matters related to vulnerable groups like the elderly. The use of an AIED event differs from prior studies that used traditional simulation methods to increase students’ attitudes toward working with older adults [23]. The computer-based virtual simulation of Millie Larson in her home permitted students to interact with the scenario at their convenience, regardless of location or time of day, if they had an internet connection. Evidence suggests there may be inherent bias in AI-driven technologies because they are built upon current evidence [24]. It is essential to explore the algorithms of the virtual simulation to ensure the virtual patient is evolving through machine learning that is sensitive to matters related to diversity, equity, and inclusion.

## 5. Limitations

There are several limitations related to this study. First, a single-site research design was utilized. Each school of nursing is unique. Thus, future studies should evaluate the effectiveness of this AIED event at other organizations. Second, the study employed a single AIED at one point in the nursing curriculum. It would be essential to explore the effectiveness of AIED at several time points across a curriculum. Repeated exposure to the simulated learning event may reveal how attitudes in older adults are or are not retained. Third, Wilson et al. concluded in their critical review of quantitative measures of attitudes toward older adults that all instruments used to date have inherent weaknesses [25]. A reliable and valid instrument to quantify attitudes toward older people has yet to be developed [26]. Magan et al. also identified the need for a current, nursing-specific measure of attitudes toward older people [9]. Finally, future studies should explore translating these findings to the knowledge of older adults in actual clinical encounters.

## 6. Conclusions

In summary, ageism within healthcare and nursing education poses a significant barrier to effective geriatric care. Addressing negative attitudes and stereotypes is crucial for fostering a compassionate and person-centered approach to caring for older adults. This research aims to contribute to the existing literature by evaluating the impact of an educational intervention on nursing students’ attitudes toward caring for older adults, as measured by the GAS. By exploring the effectiveness of the intervention, this study provides evidence to inform future educational strategies and enhance the quality of care for older adults.

## Figures and Tables

**Table 1:** Instructional teaching methods.

Phase One: Pre-AIED Event Activities Pre-class readings Ninety-minute interactive classroom session Pre-simulation medical record review Pre-simulation knowledge survey
Phase Two: AIED Event Activities AI-driven virtual non-immersive simulator experience Completion of OASIS form Create report email to multidisciplinary team
Phase Three: Post-AIED Activities Guided reflection/debrief NLN Simulation Design Scale

**Table 2:** Demographics.

Category	Frequency	Percentage
*n* = 151		
Gender		
Male	15	10%
Female	136	90%
Age Range		
18–24	74	49%
25–34	59	39%
35–44	11	7%
45–54	6	4%
55–64	1	1%
65+	0	0%
Mean	27.7	
Median	25	
Standard Deviation	7.5	

**Table 3:** Item analysis pre- and post-intervention.

Question	Pre-Intervention Mean (SD)	Post-Intervention Mean (SD)
Most old people are pleasant to be with	3.95 (0.90)	4.26 (0.76)
The federal government should reallocate money from Medicare to research on AIDS or pediatric diseases	3.45 (1.13)	3.52 (1.20)
If I have the choice, I would rather see younger patients than elderly ones	2.65 (1.23)	2.72 (1.14)
It is society’s responsibility to provide care for its elderly persons	4.15 (0.86)	4.29 (0.80)
Medicare for old people uses up too much human and material resources	4.21 (0.97)	4.29 (0.87)
As people grow older, they become less organized and more confused	3.06 (1.12)	3.45 (1.07)
Elderly patients tend to be more appreciative of the medical care I provide than are younger patients	3.28 (0.97)	3.38 (0.96)
Taking a medical history from elderly patients is frequently an ordeal	3.53 (0.99)	3.50 (1.14)
I tend to pay more attention and have more sympathy towards my elderly patients than my younger patients	2.86 (0.97)	2.97 (0.92)
Old people in general do not contribute much to society	4.47 (0.76)	4.50 (0.67)
Treatment of chronically ill old patients is hopeless	4.55 (0.69)	4.60 (0.66)
Old persons don’t contribute their fair share towards paying for their health care	4.25 (0.84)	4.33 (0.85)
In general, old people act too slow for modern society	4.27 (0.88)	4.26 (0.93)
It is interesting listening to old people’s accounts of their past experiences	4.71 (0.58)	4.68 (0.61)

## Data Availability

The data presented in this study are available on request from the corresponding author.

## Public Involvement Statement

No public involvement in any aspect of this research.

## Guidelines and Standards Statement

This manuscript was drafted against the SQUIRE-ED (Standards for Quality Improvement Reporting Excellence in Education: Publication Guidelines for Educational Improvement) for quantitative research.
